# Cognitive tasks during expectation affect the congruency ERP effects to facial expressions

**DOI:** 10.3389/fnhum.2015.00596

**Published:** 2015-10-28

**Authors:** Huiyan Lin, Claudia Schulz, Thomas Straube

**Affiliations:** ^1^Institute of Medical Psychology and Systems Neuroscience, University of MuensterMuenster, Germany; ^2^Department of Clinical Psychology, University of MuensterMuenster, Germany

**Keywords:** emotional congruency, facial expression, cognitive tasks during expectation, ERPs, N170, P3

## Abstract

Expectancy congruency has been shown to modulate event-related potentials (ERPs) to emotional stimuli, such as facial expressions. However, it is unknown whether the congruency ERP effects to facial expressions can be modulated by cognitive manipulations during stimulus expectation. To this end, electroencephalography (EEG) was recorded while participants viewed (neutral and fearful) facial expressions. Each trial started with a cue, predicting a facial expression, followed by an expectancy interval without any cues and subsequently the face. In half of the trials, participants had to solve a cognitive task in which different letters were presented for target letter detection during the expectancy interval. Furthermore, facial expressions were congruent with the cues in 75% of all trials. ERP results revealed that for fearful faces, the cognitive task during expectation altered the congruency effect in N170 amplitude; congruent compared to incongruent fearful faces evoked larger N170 in the non-task condition but the congruency effect was not evident in the task condition. Regardless of facial expression, the congruency effect was generally altered by the cognitive task during expectation in P3 amplitude; the amplitudes were larger for incongruent compared to congruent faces in the non-task condition but the congruency effect was not shown in the task condition. The findings indicate that cognitive tasks during expectation reduce the processing of expectation and subsequently, alter congruency ERP effects to facial expressions.

## Introduction

From an evolutionary perspective, expecting the emotional significance of an upcoming event on the basis of environmental cues may help an individual in preparing adaptive reactions to potentially threatening situations (Nitschke et al., 2006; Galli et al., 2011). Nevertheless, in an ever-changing environment, the events sometimes turn out to be incongruent with our expectations (e.g., Schnider et al., 2007; Vachon et al., 2012; Barbalat et al., 2013) and the adaptive task is therefore to detect such expectancy incongruent events. This is, for example, relevant in the context of processing facial expressions, as some facial expressions (i.e., fearful) represent potential threat. Therefore, the processing of facial expression depending on expectancy congruency is a growing field of research in human neuroscience (e.g., Hirai et al., 2008; Li et al., 2008; Herbert et al., 2013; Hietanen and Astikainen, 2013).

ERPs are ideal to investigate rapid changes in neural responses during the perception of stimuli which are congruent or incongruent with individuals' expectations. Several ERP components have been shown to be modulated by expectancy congruency, specifically the expectancy congruency of facial expressions. Concerning an early ERP time range, the N170 seems to be of great importance regarding to expectancy congruency to facial expressions, as this component is thought to be sensitive to faces (Bentin et al., 2007). The N170, which peaks around 170 ms post-stimulus and is maximal at occipito-temporal scalp sites, is associated with face encoding (e.g., Eimer and McCarthy, 1999; Eimer, 2000a,b). The N170 is found to be larger for faces which are congruent compared to incongruent with an observer's norms or expectations (e.g., typical vs. atypical faces; Halit et al., 2000; Freeman et al., 2010). Regarding to the expectancy congruency of facial expressions, the N170 is larger for congruent as compared to incongruent facial expressions (Hietanen and Astikainen, 2013).

In addition, the P3 component (overlapping with late positive potentials, LPP), a positive deflection starting at around 300 ms after stimulus presentation over parietal scalp sites, is associated with the attention allocation (Hajcak et al., 2006; Olofsson et al., 2008). The P3 has been repeatedly observed to be enhanced by stimuli that are incongruent with expectations (e.g., Delplanque et al., 2005; Volpe et al., 2007). With regard to facial expressions, P3 amplitudes for sad faces were found to be more positive after emotionally incongruent as compared to congruent primes (Hietanen and Astikainen, 2013).

The question arises whether the congruency ERP effects to emotional stimuli (e.g., facial expressions) might be modulated if stimulus expectation is manipulated by cognitive tasks. Cognitive tasks during presentations of emotional stimuli are supposed to alter emotional responses by directing the attention away from emotional stimuli to given cognitive tasks (Ochsner and Gross, 2005; McRae et al., 2010). Studies have shown that such cognitive tasks reduced emotional responses as well as corresponding neural activity elicited by the emotional stimuli (e.g., McRae et al., 2010; Kanske et al., 2011). More importantly, some studies found that cognitive tasks performed during expectation of emotional stimuli reduced the processing of expectation (Del Percio et al., 2006; Erk et al., 2006; Kalisch et al., 2006; Kanske et al., 2010). However, it is as yet unknown whether cognitive tasks during expectation change the perception of emotionally incongruent as compared to congruent stimuli.

The present study aimed to investigate effects of cognitive tasks during expectation on ERP responses to emotionally incongruent vs. congruent faces. To address this issue, participants were asked to perform a cue—face paradigm (S1–S2 task). In the cue—face paradigm, a probabilistic stimulus (S1) indicated a specific expression (either fearful or neutral) of an upcoming face (S2). In some trials, the prediction was violated and the other expression was presented. In half of the trials, participants had to solve a cognitive task in which a target letter was embedded in an array of distractor letters between the cue and the face; while there was no task in the other half. Based on the above-mentioned studies, we expected that for the non-task trials, incongruent compared to congruent faces would evoke smaller N170 but larger P3, especially for fearful faces; but such congruency effects would be altered by the cognitive task during expectation.

## Methods

### Participants

Twenty-four participants were recruited in Muenster via advertisement and were paid 10 Euros for participation. Two participant were excluded from the statistical analysis because of excessive artifacts in the EEG signal, resulting in a total of 22 participants (20–43 years old, *M* = 26.70, *SD* = 6.13; 14 females). All participants were right-handed as determined by the Edinburgh Handedness Inventory (Oldfield, 1971). All participants had normal or corrected-to-normal vision and none of the participants reported a history of neurological illness. The study was conducted in accordance with standard ethical guidelines as defined in the Declaration of Helsinki and written informed consent was obtained prior the testing. The study was approved by the ethics committee of the University of Muenster.

### Stimuli

**Figure 1:**
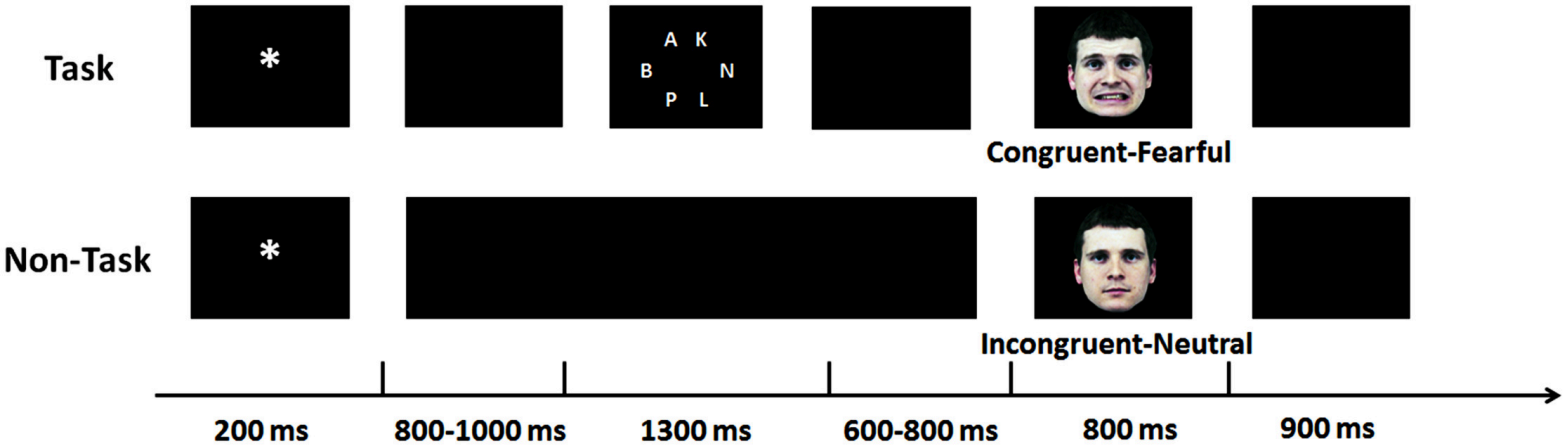
**Experimental procedure**. A cue-face paradigm was performed with a cognitive task between the cue and the face (the upper panel) and without (the lower panel).

We selected 80 digitized color photographs from the Karolinska Directed Emotional Faces (KDEF, Lundqvist et al., 1998) database. The face stimuli portrayed 40 individuals (20 females), each showing two expressions (fearful or neutral). Using Adobe Photoshop CS6, we adjusted the size of raw pictures to 5.37° × 7.15° (horizontal × vertical), removed the neck, shoulders and distant hair, and matched the pictures in luminance, contrast, hue and color. Examples of the face stimuli are shown in Figure 1.

### Procedure

After informed consent had been given and handedness had been determined, participants were told to perform the cognitive task as well as the cue—face task. For the cognitive task, they were asked to indicate which target letter they detected during simultaneous presentation of six letters on the screen by button press. For the cue—face task, participants were told that the face was always presented with a preceding cue, and they were informed of the meaning of the cue (e.g., the symbol “^*^” is often followed by a fearful face but sometimes also by a neutral face). Participants were instructed to view the cues and faces during their presentations and to indicate the expression of the face during presentation of the face or the following blank. The instructions for both tasks emphasized speed as well as accuracy. Participants were told to respond to the facial expression by the “F” and the “J” key with the left and the right index finger, respectively, and to the target letter by the “3” and the “9” key with the left and the right middle finger, respectively. Participants were required to rest their fingers on these keys even when they were not required to press the keys (e.g., the keys “3” and “9” in the cue—face task without the cognitive task). For each of the task, the assignments of responses were counterbalanced across participants.

Stimuli were presented using E-Prime 2.0 software (Psychology Software Tools, Inc., Pittsburgh, PA, USA) on a black screen in the center of a 15″ monitor with a screen resolution of 1280 by 1024 pixel. Viewing distance was approximately 80 cm. All stimuli were presented against a dark background.

Figure 1 illustrates the time course of stimulus presentation for one trial. Each trial started with a white cue (“^*^” or “^#^,” 0.72° × 0.72°) for 200 ms, followed by a blank screen for 800 to 1000 ms (*M* = 900 ms). For half of the participants, the cue “^*^” was mostly (75%) followed by a fearful face but occasionally (25%) by a neural face and the cue “^#^” was mostly (75%) followed by a neutral face but occasionally (25%) by a fearful face; for the other half of the participants, the meaning of the cues was switched. The face was presented for 800 ms. Another blank screen of 900 ms was presented before the next trial started.

After the blank screen followed by the cue, a cognitive task had to be solved in half of the trials, followed by another blank screen for 600–800 ms (*M* = 700 ms). For the cognitive task, six white letters appeared at equally-spaced intervals on the circumference of an array with a diameter of 5.01° for 1300 ms. The letters comprised a target (“N” or “X,” 0.48° × 0.48°) which was presented equally often in each potential location and five non-targets (0.48° × 0.48°) which were selected randomly from the other 24 letters of the Latin script (A-Z, except N and X). In the other half of the trials, the blank screen was presented continually, matching the duration of the cognitive task and the blank screen followed by the task. Note that the duration of the sequences varied in the same manner in the non-task and the task condition. Task and non-task conditions were completed as two separate blocks, and block order was counterbalanced and randomized across participants.

Participants were asked whether they remembered the meaning of the cue after the task and the non-task block, respectively (e.g., if you see the “^*^,” was the following face mostly neutral or fearful?). All participants answered correctly for both the task and the non-task block, indicating that they did not forget the meaning of the cue during the experiment. The task comprised a total of 640 trials, with 120 for congruent and 40 for incongruent faces, for each facial expression and each block. Additionally, there were 20 practice trials before the actual experiment started. The complete experiment lasted for about an hour.

### Behavioral data

Response times (RTs) and accuracy (ACC) of button presses in the time range from the onset of the face to the offset of the following blank were recorded. For the analysis of RTs, trials only with correct responses were included.

### EEG recording

EEG was continuously recorded using a 32-channel BioSemi Active II System (BioSemi, Amsterdam, Netherlands). Thirty-two Ag/AgCl Active electrodes were placed on the scalp by means of an elastic head cap (BioSemi, Amsterdam), according to the 10–20 International System (FP1, FP2, F7, F3, Fz, F4, F8, FC5, FC1, FC2, FC6, T7, C3, Cz, C4, T8, TP9, CP1, CP2, TP10, P7, P9, P3, Pz, P4, P8, P10, PO9, O1, Oz, O2, PO10). The BioSemi System uses an active electrode (CMS—common mode sense) and a passive electrode (DRL - driven right leg) to form a feedback loop with two additional electrodes instead of ground and reference (please see http://www.biosemi.com/faq/cms&drl.htm). The horizontal electrooculogram (EOG) was recorded from two electrodes at the outer canthi of both eyes, and the vertical EOG was recorded bipolarly from two electrodes above and below the right eye to monitor eye blinks and movements. All signals were digitized with a sampling rate of 2048 Hz and a low-pass fifth order sinc filter with a half-power cutoff at 100 Hz. Impedances were generally kept below 10 kΩ.

During offline processing, ocular artifacts were automatically corrected by means of BESA 6.0 software (www.BESA.de). The continuous EEGs were then segmented from −200 to 800 ms relative to onset of the face stimuli, using the first 200 ms of signal for baseline correction. Artifact rejection was performed based on an amplitude threshold of 120 μV, a gradient criterion of 75 μV and low sig criterion of 0.01 μV (the default parameter of the BESA 6.0 artifact rejection tool). Trials were averaged separately for each channel and experimental condition. Averaged ERPs were recalculated to average reference, excluding vertical and horizontal EOG channels, and ERPs were then low-pass filtered at 40 Hz (Butterworth zero phase shift). The mean, minimum and maximum number of trials are presented in Table 1.

**Table 1:** *****Mean, minimum*** and ***maximum*** number of trials for all the experimental conditions**.

	**Neutral faces**						**Fearful faces**					
	**Non-task**			**Task**			**Non-task**			**Task**		
	***M***	***Min***	***Max***	***M***	***Min***	***Max***	***M***	***Min***	***Max***	***M***	***Min***	***Max***
Congruent	108.41	87	120	107.77	84	119	106.82	87	120	107.95	88	119
Incongruent	35.82	28	40	36.45	31	40	35.77	29	40	36.73	30	40

ERPs were quantified using mean amplitudes for occipital-temporal N170 (130–180 ms) and parietal P3 (450–650 ms), all relative to a baseline lasting from −200 to 0 ms. N170 and P3 were measured at electrodes PO9/PO10 and P3/Pz/P4, respectively. The time-window for N170 was chosen on a basis of peaks identified in the grand waveforms across all conditions (155 ms) and the time window for P3 was selected based on previous studies (Delplanque et al., 2005; Volpe et al., 2007) and visual inspection of the grand waveforms. Electrodes of interest were selected based on visual inspection of the grand means and previous studies (Delplanque et al., 2005; Latinus and Taylor, 2006; Volpe et al., 2007; Herbert et al., 2013).

### Data analysis

**Figure 2:**
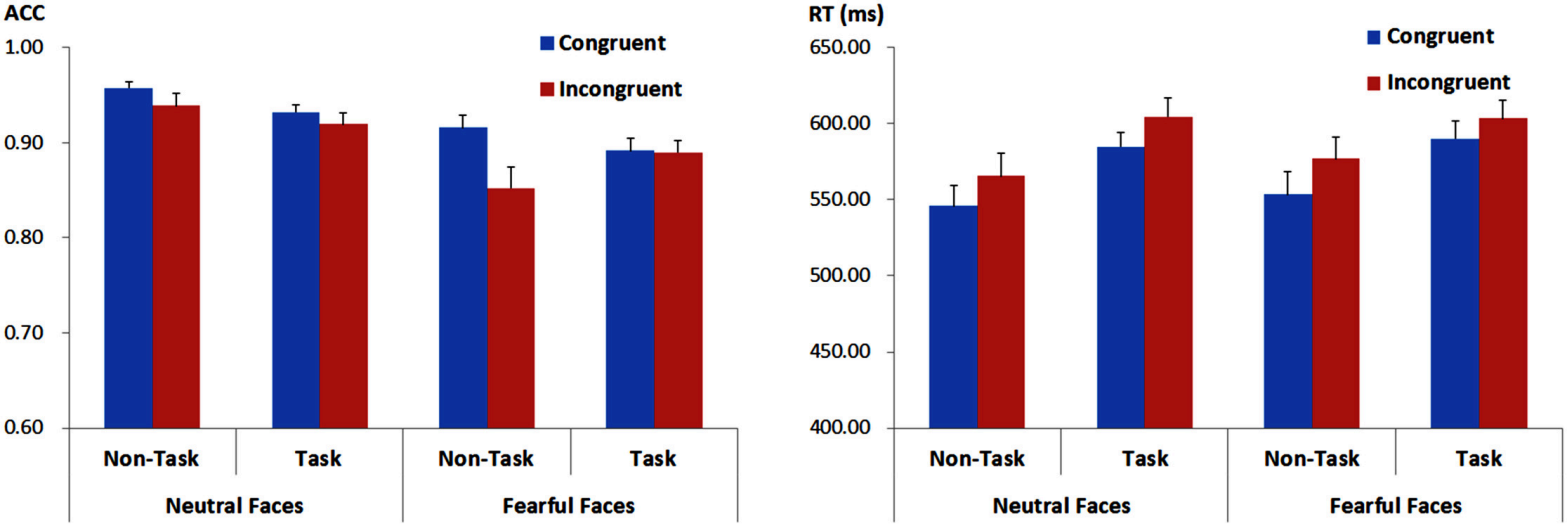
**ACC (the left panel) and RTs (the right panel) for recognition of facial expressions for each experimental condition**. Vertical lines indicate the standard error of the mean.

For behavioral ACC and RTs in recognition of facial expressions, we performed 2 × 2 × 2 analyses of variance (ANOVAs) with “manipulation of expectation” (task and non-task), facial expression (fearful and neutral) and congruency (congruent and incongruent) as within-subject factors. *Mean* and *SE* of ACC and RTs across conditions are presented in Figure 2 and Tables 2, 3.

**Table 2:** *****Mean*** of ACC and its ***SE*** for all the experimental conditions**.

	**Neutral faces**				**Fearful faces**			
	**Non-task**		**Task**		**Non-task**		**Task**	
	***M***	***SE***	***M***	***SE***	***M***	***SE***	***M***	***SE***
Congruent	0.96	0.01	0.93	0.01	0.92	0.01	0.89	0.01
Incongruent	0.94	0.01	0.92	0.01	0.85	0.02	0.89	0.01

**Table 3:** *****Mean*** of RTs (ms) and its ***SE*** for all the experimental conditions**.

	**Neutral faces**				**Fearful faces**			
	**Non-task**		**Task**		**Non-task**		**Task**	
	***M***	***SE***	***M***	***SE***	***M***	***SE***	***M***	***SE***
Congruent	548.81	12.98	588.19	9.48	556.39	14.97	596.16	12.26
Incongruent	569.53	15.02	605.83	11.94	581.15	13.73	607.80	12.21

**Figure 3:**
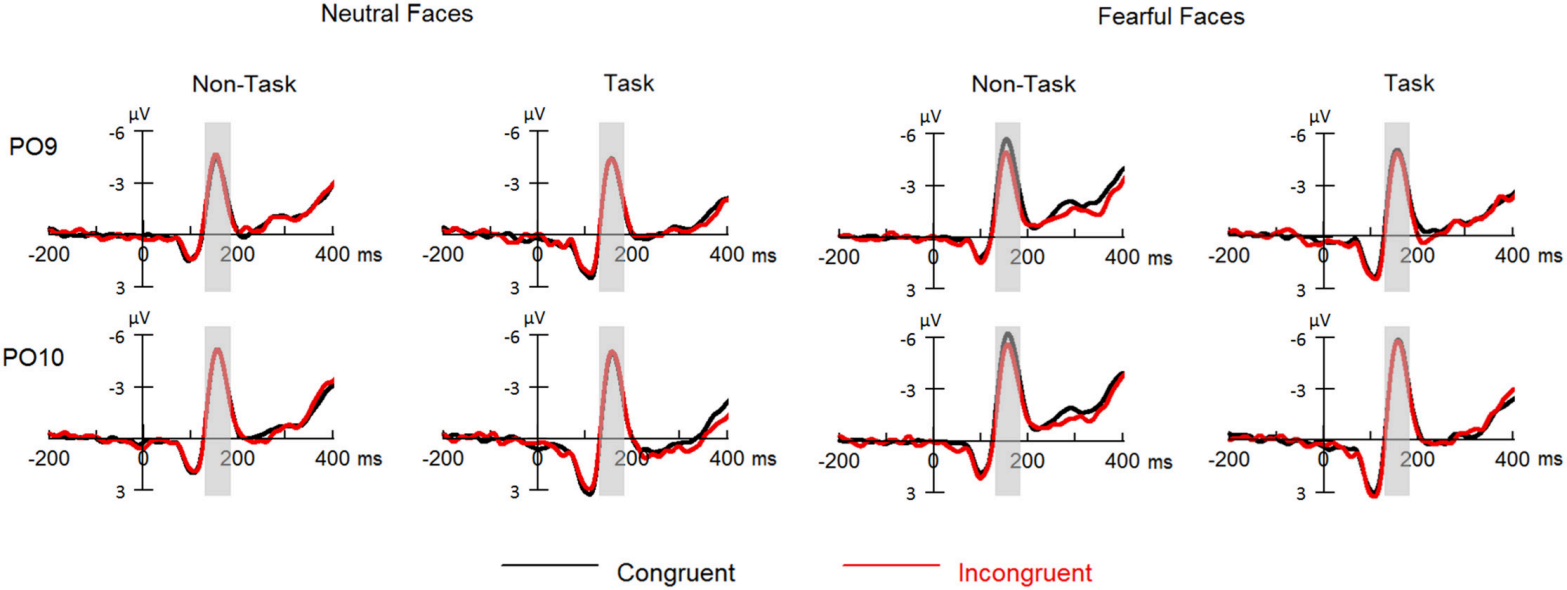
**ERPs at parietal- occipital electrodes (PO9 and PO10) for all the experimental conditions**. Shaded areas correspond to the analysis window for the N170 (130–180 ms).

**Figure 4:**
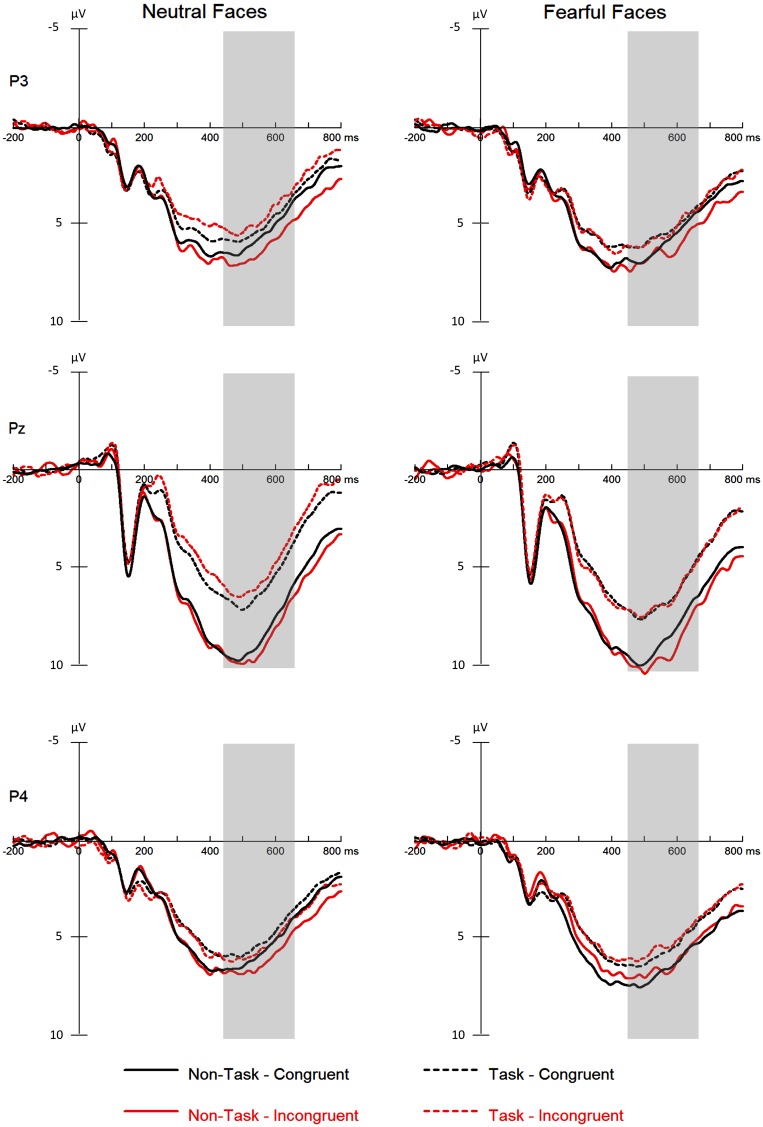
**ERPs at the parietal electrodes (P3, Pz, and P4) for all the experimental conditions**. Shaded areas correspond to the analysis window for the P3 (450–650 ms).

**Figure 5:**
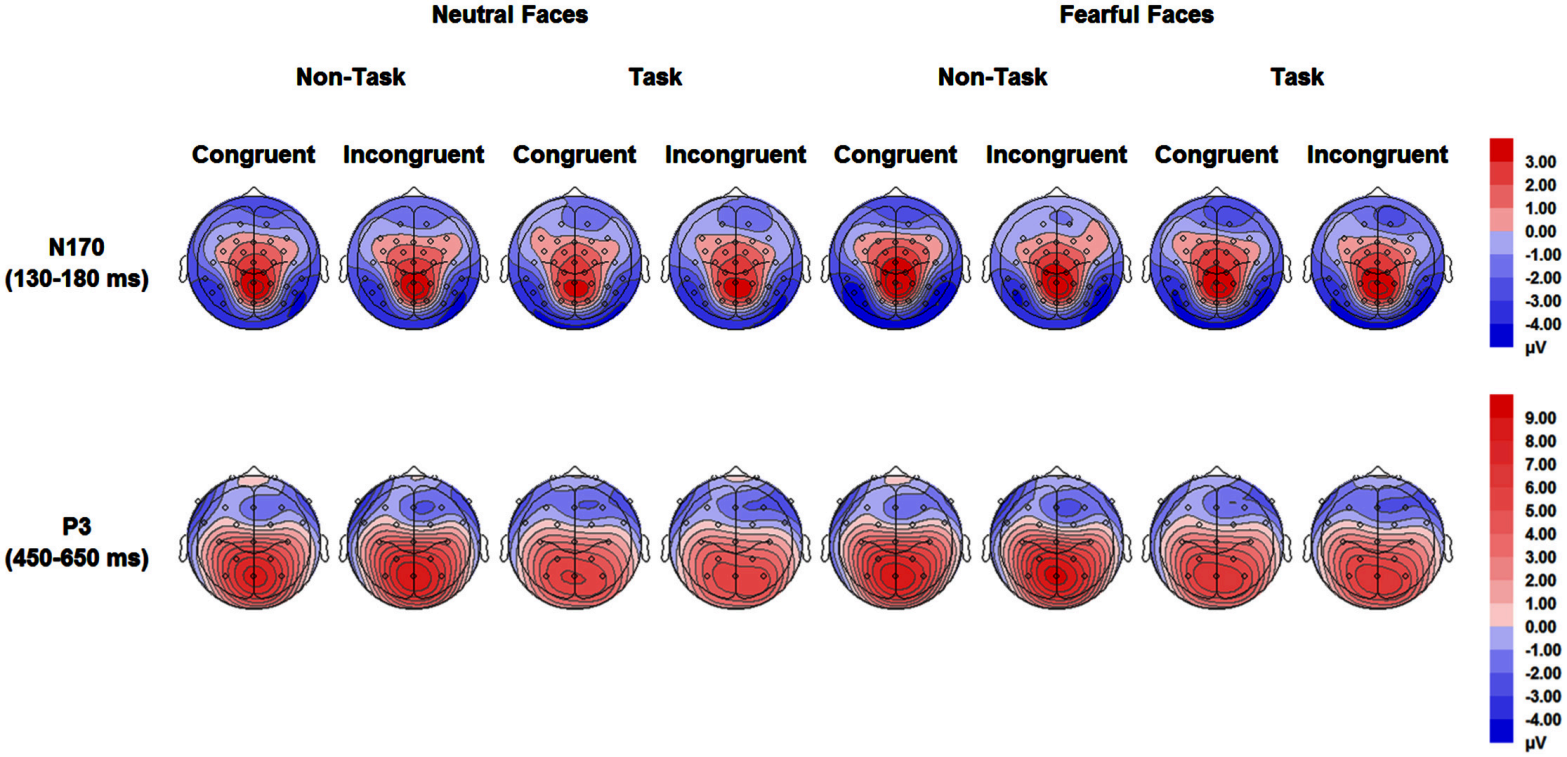
**Topographical maps based on mean amplitudes of N170 (130–180 ms) and P3 (450–650 ms) for all experimental conditions**.

For ERPs, repeated measures ANOVAs with within-subject factors “manipulation of expectation” (task and non-task), facial expression (fearful and neutral) and congruency (congruent and incongruent) were performed separately for the N170 and the P3. The analysis for the N170 and the P3 included hemisphere (left: PO9 and right: PO10) and electrode (P3, Pz, P4), respectively, as additional within-subject factors. Grand-average waveforms of the N170 and the P3 are presented in Figures 3, 4, respectively. Topographical maps for these two components are presented in Figure 5. *Mean* and *SE* of the N170 and the P3 across conditions are presented in Tables 4, 5, respectively.

**Table 4:** *****Mean*** of N170 amplitude (μV) and its ***SE*** for all the experimental conditions**.

		**Neutral faces**				**Fearful faces**			
		**Non-task**		**Task**		**Non-task**		**Task**	
		***M***	***SE***	***M***	***SE***	***M***	***SE***	***M***	***SE***
PO9	Congruent	−2.92	0.63	−2.69	0.68	−4.07	0.65	−3.07	0.73
	Incongruent	−3.00	0.72	−2.56	0.80	−3.29	0.66	−3.04	0.76
PO10	Congruent	−4.09	0.81	−3.56	0.73	−5.06	0.80	−4.24	0.75
	Incongruent	−4.10	0.83	−3.57	0.74	−4.45	0.82	−4.20	0.78

**Table 5:** *****Mean*** of P3 amplitude (μV) and its ***SE*** for all the experimental conditions**.

		**Neutral faces**				**Fearful faces**			
		**Non-task**		**Task**		**Non-task**		**Task**	
		***M***	***SE***	***M***	***SE***	***M***	***SE***	***M***	***SE***
P3	Congruent	5.55	0.53	4.96	0.65	6.05	0.53	5.32	0.59
	Incongruent	6.27	0.59	4.55	0.63	6.37	0.52	5.39	0.58
Pz	Congruent	8.40	0.88	5.87	0.90	8.79	1.04	6.58	0.90
	Incongruent	8.81	0.91	5.24	0.97	9.25	1.00	6.53	0.86
P4	Congruent	5.56	0.67	4.96	0.67	6.55	0.72	5.51	0.74
	Incongruent	6.14	0.72	5.23	0.79	6.29	0.76	5.19	0.76

Greenhouse-Geisser and Bonferroni correction were applied to correct degrees of freedom and *p*-values of repeated measurements and *post-hoc* tests, respectively, where appropriate. A probability level of *p* < 0.05 was considered statistically significant.

## Results

### Behavioral results

#### ACC

There were main effects of facial expression [*F*_(1, 21)_ = 19.65, *p* < 0.001, $\eta_{p}^{2}=0.48$] and congruency [*F*_(1, 21)_ = 10.85, *p* = 0.003, $\eta_{p}^{2}=0.34$]. ACC was higher for neutral as compared to fearful faces and for congruent as compared to incongruent faces. However, the main effect of “manipulation of expectation” was not significant [*F*_(1, 21)_ = 0.74, *p* = 0.401, $\eta_{p}^{2}=0.03$].

The interaction between “manipulation of expectation” and facial expression was significant [*F*_(1, 21)_ = 7.30, *p* = 0.013, $\eta_{p}^{2}=0.26$]. Further analyses showed higher ACC for neutral than fearful faces in both the task [*F*_(1, 21)_ = 10.30, *p* = 0.004, $\eta_{p}^{2}=0.33$] and the non-task condition [*F*_(1, 21)_ = 21.81, *p* < 0.001, $\eta_{p}^{2}=0.51$], although to different extents. The two-way interaction between “manipulation of expectation” and congruency also reached statistical significance [*F*_(1, 21)_ = 4.49, *p* = 0.046, $\eta_{p}^{2}=0.18$]. In the non-task condition, the ACC was higher for congruent as compared to incongruent faces [*F*_(1, 21)_ = 9.52, *p* = 0.006, $\eta_{p}^{2}=0.31$]; while the effect of congruency was not significant in the task condition [*F*_(1, 21)_ = 0.94, *p* = 0.344, $\eta_{p}^{2}=0.04$]. However, there was no interaction between facial expression and congruency [*F*_(1, 21)_ = 1.81, *p* = 0.192, $\eta_{p}^{2}=0.08$].

The effect above was further qualified by a three-way interaction [*F*_(1, 21)_ = 9.89, *p* = 0.005, $\eta_{p}^{2}=0.32$]. Separate analysis for each facial expression showed that for fearful faces, the analysis showed a main effect of congruency, with higher ACC for congruent as compared to incongruent faces [*F*_(1, 21)_ = 7.69, *p* = 0.011, $\eta_{p}^{2}=0.27$], but the main effect of “manipulation of expectation” was not significant [*F*_(1, 21)_ = 0.45, *p* = 0.509, $\eta_{p}^{2}=0.02$]. More importantly, the interaction between “manipulation of expectation” and congruency was also significant [*F*_(1, 21)_ = 10.38, *p* = 0.004, $\eta_{p}^{2}=0.33$]. In the non-task condition, the ACC was higher for congruent as compared to incongruent faces [*F*_(1, 21)_ = 11.64, *p* = 0.003, $\eta_{p}^{2}=0.36$]; while in the task condition, the congruency effect was not significant [*F*_(1, 21)_ = 0.03, *p* = 0.861, $\eta_{p}^{2}$ < 0.01]. For neutral faces, the ACC was overall higher for congruent as compared to incongruent faces [*F*_(1, 21)_ = 5.28, *p* = 0.032, $\eta_{p}^{2}=0.20$], but the main effect of “manipulation of expectation” [*F*_(1, 21)_ = 3.89, *p* = 0.062, $\eta_{p}^{2}=0.16$] and the interaction between congruency and “manipulation of expectation” were not significant [*F*_(1, 21)_ = 0.12, *p* = 0.731, $\eta_{p}^{2}=0.01$]. However, Figure 2 implies that the difference for congruent compared to incongruent neutral faces seems to be too small in both the task and the non-task condition. Consequently, we further analyzed the congruency effect in the task and the non-task condition, respectively. Indeed, the congruency effect was not significant in both conditions [the non-task condition: *F*_(1, 21)_ = 2.62, *p* = 0.121, $\eta_{p}^{2}=0.11$; the task condition: *F*_(1, 21)_ = 1.51, *p* = 0.232, $\eta_{p}^{2}=0.07$].

#### RTs

For RTs, there were main effects of “manipulation of expectation” [*F*_(1, 21)_ = 15.57, *p* = 0.001, $\eta_{p}^{2}=0.43$) and congruency [*F*_(1, 21)_ = 20.28, *p* < 0.001, $\eta_{p}^{2}=0.49$]. RTs were longer in the task as compared to the non-task condition and for incongruent as compared to congruent faces. Other main effects or interactions were not significant [facial expression: *F*_(1, 21)_ = 1.11, *p* = 0.305, $\eta_{p}^{2}=0.05$; “manipulation of expectation” × facial expression: *F*_(1, 21)_ = 0.47, *p* = 0.501, $\eta_{p}^{2}=0.02$; “manipulation of expectation” × congruency: *F*_(1, 21)_ = 1.05, *p* = 0.317, $\eta_{p}^{2}=0.05$; facial expression × congruency: *F*_(1, 21)_ = 0.03, *p* = 0.873, $\eta_{p}^{2}$ < 0.01; “manipulation of expectation” × facial expression × congruency: *F*_(1, 21)_ = 0.82, *p* = 0.376, $\eta_{p}^{2}=0.04$].

### ERP results

#### N170 component

Overall, N170 amplitude was larger for fearful as compared to neutral faces [*F*_(1, 21)_ = 34.42, *p* < 0.001, $\eta_{p}^{2}=0.62$] and for congruent as compared to incongruent faces [*F*_(1, 21)_ = 5.30, *p* = 0.032, $\eta_{p}^{2}=0.20$].

These effects were qualified by a significant three-way interaction among “manipulation of expectation,” facial expression and congruency [*F*_(1, 21)_ = 6.37, *p* = 0.020, $\eta_{p}^{2}=0.23$]. Separate analysis for each emotion showed that for neutral faces, no main effects or interaction reached statistical significance [“manipulation of expectation”: *F*_(1, 21)_ = 1.64, *p* = 0.215, $\eta_{p}^{2}=0.07$; congruency: *F*_(1, 21)_ < 0.01, *p* = 0.977, $\eta_{p}^{2}$ < 0.01; “manipulation of expectation” × congruency: *F*_(1, 21)_ = 0.33, *p* = 0.570, $\eta_{p}^{2}=0.02$]. For fearful faces, the N170 amplitude was significantly larger for congruent as compared to incongruent faces [*F*_(1, 21)_ = 6.84, *p* = 0.016, $\eta_{p}^{2}=0.25$], but the main effect of “manipulation of expectation” was not significant [*F*_(1, 21)_ = 3.32, *p* = 0.083, $\eta_{p}^{2}=0.14$]. More importantly, the analysis showed a significant interaction between “manipulation of expectation” and congruency [*F*_(1, 21)_ = 8.18, *p* = 0.009, $\eta_{p}^{2}=0.28$]. Congruent as compared to incongruent faces elicited greater amplitudes in the non-task condition [*F*_(1, 21)_ = 16.41, *p* = 0.001, $\eta_{p}^{2}=0.44$]; while the congruency effect was not significant in the task condition [*F*_(1, 21)_ = 0.04, *p* = 0.853, $\eta_{p}^{2}$ < 0.01].

However, other main effects or interaction did not reach statistical significance [“manipulation of expectation”: *F*_(1, 21)_ = 2.57, *p* = 0.124, $\eta_{p}^{2}=0.11$; hemisphere: *F*_(1, 21)_ = 3.53, *p* = 0.074, $\eta_{p}^{2}=0.14$; “manipulation of expectation” × facial expression: *F*_(1, 21)_ = 0.65, *p* = 0.429, $\eta_{p}^{2}=0.03$; facial expression × congruency: *F*_(1, 21)_ = 2.30, *p* = 0.144, $\eta_{p}^{2}=0.10$; “manipulation of expectation” × congruency: *F*_(1, 21)_ = 4.07, *p* = 0.057, $\eta_{p}^{2}=0.16$; “manipulation of expectation” × hemisphere: *F*_(1, 21)_ = 0.04, *p* = 0.849, $\eta_{p}^{2}$ < 0.01; facial expression × hemisphere: *F*_(1, 21)_ = 0.73, *p* = 0.403, $\eta_{p}^{2}=0.03$; congruency × hemisphere: *F*_(1, 21)_ = 0.28, *p* = 0.605, $\eta_{p}^{2}=0.01$; “manipulation of expectation” × facial expression × hemisphere: *F*_(1, 21)_ = 2.14, *p* = 0.158, $\eta_{p}^{2}=0.93$; “manipulation of expectation” × congruency × hemisphere: *F*_(1, 21)_ < 0.01, *p* = 0.973, $\eta_{p}^{2}$ < 0.01; facial expression × congruency × hemisphere: *F*_(1, 21)_ = 0.04, *p* = 0.849, $\eta_{p}^{2}$ < 0.01; “manipulation of expectation” × facial expression × congruency × hemisphere: *F*_(1, 21)_ = 1.40, *p* = 0.250, $\eta_{p}^{2}=0.06$].

#### P3 component

The analysis yielded significant main effects of “manipulation of expectation” [*F*_(1, 21)_ = 23.46, *p* < 0.001, $\eta_{p}^{2}=0.53$], facial expression [*F*_(1, 21)_ = 7.94, *p* = 0.010, $\eta_{p}^{2}=0.27$] and electrode [*F*_(2, 42)_ = 10.41, *p* < 0.001, $\eta_{p}^{2}=0.33$]. Overall, the P3 was more positive in the non-task as compared to the task condition, for fearful as compared to neutral faces and for electrode Pz as compared to P3 (*p* = 0.004) and P4 (*p* = 0.001).

The interaction between “manipulation of expectation” and electrode [*F*_(2, 33)_ = 6.98, *p* = 0.005, $\eta_{p}^{2}=0.25$] was significant. The P3 was more pronounced in the non-task as compared to the task condition at all electrodes, though to different extent [at P3: *F*_(1, 21)_ = 9.22, *p* = 0.006, $\eta_{p}^{2}=0.31$; at Pz: *F*_(1, 21)_ = 23.12, *p* < 0.001, $\eta_{p}^{2}=0.52$; at P4: *F*_(1, 21)_ = 4.55, *p* = 0.045, $\eta_{p}^{2}=0.18$].

In addition, the interaction between “manipulation of expectation” and congruency was significant [*F*_(1, 21)_ = 7.44, *p* = 0.013, $\eta_{p}^{2}=0.26$]. Amplitudes were larger for incongruent relative to congruent faces in the non-task condition [*F*_(1, 21)_ = 4.37, *p* = 0.049, $\eta_{p}^{2}=0.17$], whereas no difference was found in the task condition [*F*_(1, 21)_ = 2.03, *p* = 0.169, $\eta_{p}^{2}=0.09$].

We did not find the main effect of congruency [*F*_(1, 21)_ = 0.71, *p* = 0.409, $\eta_{p}^{2}=0.03$], the two-way interaction “manipulation of expectation” × facial expression [*F*_(1, 21)_ = 0.31, *p* = 0.582, $\eta_{p}^{2}=0.02$], facial expression × congruency [*F*_(1, 21)_ = 0.54, *p* = 0.470, $\eta_{p}^{2}=0.03$], facial expression × electrode [*F*_(2, 42)_ = 0.73, *p* = 0.490, $\eta_{p}^{2}=0.03$] or congruency × electrode [*F*_(2, 42)_ = 0.26, *p* = 0.770, $\eta_{p}^{2}=0.01$], the three-way interaction of “manipulation of expectation” × facial expression × congruency [*F*_(1, 21)_ = 1.89, *p* = 0.183, $\eta_{p}^{2}=0.08$], facial expression × congruency × electrode [*F*_(2, 33)_ = 2.97, *p* = 0.077, $\eta_{p}^{2}=0.12$] or “manipulation of expectation” × facial expression × electrode [*F*_(2, 39)_ = 1.90, *p* = 0.165, $\eta_{p}^{2}=0.08$] or the four-way interaction [*F*_(2, 42)_ = 0.38, *p* = 0.684, $\eta_{p}^{2}=0.02$].

## Discussion

The present study investigated whether the congruency effects to facial expressions are modulated by cognitive tasks during expectation. Results on accuracy showed that cognitive tasks during expectation modulated the congruency effect for fearful faces; the accuracy was lower for incongruent as compared to congruent fearful faces in the non-task condition but the congruency effect was not significant in the task condition. More importantly, ERP results showed that for fearful faces, the congruency effect was modulated by the cognitive task during expectation in the N170; N170 amplitudes were smaller for fearful faces after the incongruent vs. the congruent cue in the non-task condition but such a congruency effect was not evident in the task condition. For the P3 component, the cognitive task during expectation generally altered the congruency effect, regardless of facial expressions; emotionally incongruent as compared to congruent faces were larger in P3 amplitude in the non-task condition but such a congruency effect was not shown in the task condition. These ERP results suggest that cognitive tasks during expectation modify the congruency ERP effects to facial expressions.

Expectation allows participants to prepare for an upcoming stimulus in order to modulate the processing of the stimulus after it occurs (Onoda et al., 2007; Grupe and Nitschke, 2011). Research suggests that cognitive tasks during expectation of emotional stimuli reduce the processing of expectation (Del Percio et al., 2006; Erk et al., 2006; Kalisch et al., 2006). For example, expecting negative events produced greater activation in certain brain regions (e.g., amygdala as well as the anterior rostral medial prefrontal cortex) associated with negative emotions than did expecting neutral events without any cognitive tasks during expectation (Herwig et al., 2007). However, the differential activity was reduced when cognitive tasks were performed during expectation (Erk et al., 2006). Accordingly, in our present study, cognitive tasks during expectation may reduce the processing of expectation and subsequently, alter the effects of emotional congruency.

Behavioral data showed that cognitive tasks during expectation modulated the congruency effect for fearful faces in accuracy; the accuracy was higher for fearful faces after the congruent as compared to the incongruent cue without cognitive tasks during expectation, but such congruency effect was not evident when cognitive tasks were performed during expectation. Many studies have indicated that while expectation allows individuals to prepare the perception for the upcoming stimulus, expectation interferes with the perception if the stimulus is incongruent with the expectation (e.g., Pourtois et al., 2004; Kanske et al., 2011). Therefore, the present results indicate that cognitive tasks reduce the perception preparation during the expectation phase and thereby, alter the difference between congruent and incongruent fearful faces in perception accuracy. However, for neutral faces, the accuracy was similar after the congruent compared to the incongruent cue in both the non-task and the task condition. Expectation of fearful as compared to neutral stimuli allows individuals to enhance vigilance (Böcker et al., 2001; Babiloni et al., 2004). Neutral faces expected as fearful (incongruent neutral faces) compared with those expected as neutral (congruent neutral faces) may be therefore perceived precisely and did not reduce the perception accuracy. As incongruent compared to congruent neutral faces were similar in accuracy in the non-task condition, it makes sense that cognitive tasks during expectation did not modulate the accuracy effect of congruency for neutral faces.

For the RTs, however, the congruency effect was not modulated by cognitive tasks during expectation; RTs were slower for incongruent compared to congruent faces in both the non-task and the task condition. The reason that the congruency effect was observed in the task condition may be that expectation was not totally prevented during the expectancy intervals. In the task condition, while expectation was prevented during solving the cognitive task, it was not the case after the task. Participants can expect the facial expressions immediately before the faces (at least 600 ms). This time range has been indicated to be related to action preparation to the upcoming stimulus (e.g., Rohrbaugh et al., 1976; Sakamoto et al., 2009; Lin et al., 2014), possibly resulting in failing to reduce the congruency effect.

Regarding to ERPs, the N170 is often thought to be a marker of face encoding (e.g., Eimer and McCarthy, 1999; Eimer, 2000a,b). The amplitudes were found to be larger for faces congruent compared with incongruent with an observer's norms/expectations (Halit et al., 2000; Freeman et al., 2010). Regarding to the expectancy congruency to facial expressions, Hietanen and Astikainen (2013) found that the N170 amplitudes were larger for facial expressions after congruent compared to incongruent primes. Therefore, the present results suggest that cognitive tasks alter the congruency effect for fearful faces related to face encoding.

One possible explanation regarding to the effect of cognitive tasks is that cognitive tasks reduce fearful evaluation during the expectation of fearful expressions. The spreading activation account indicates that expecting the emotional content (e.g., threat) of a stimulus automatically activates the corresponding emotion evaluation during the expectation phase and therefore, strengthens the encoding of the emotionally congruent stimulus during the perception phase (Fazio, 2001; Klauer and Musch, 2003). Therefore, it is likely that cognitive tasks reduce the automatic activation of fearful evaluation during the expectation phase and, thus modify the difference between congruent and incongruent fearful faces regarding to the encoding. In terms of neural activity, expecting the emotional content of a face may activate certain brain regions (i.e., inferior occipital gyrus, fusiform gyrus, and superior temporal sulcus), leading to facilitated activation during the presentation of the face (Hietanen and Astikainen, 2013). As these regions are thought to be the source of N170, cognitive tasks may prevent the activation in such brain regions during the expectation phase and therefore, alter the congruency N170 effect.

For neutral faces, however, we did not find that cognitive tasks during expectation modulated the N170 effect of expectancy congruency. In fact, there was no congruency N170 effect in both the non-task and the task condition. According to previous studies (e.g., Eimer and McCarthy, 1999; Eimer, 2000a,b), our results indicate that the encoding of congruent compared with incongruent neutral faces is similar in both the non-task and the task condition. While expectation of emotional stimuli activates the corresponding emotion (and/or certain brain regions associated with the stimuli) in order to facilitate the encoding of emotionally congruent stimuli (Fazio, 2001; Klauer and Musch, 2003; Hietanen and Astikainen, 2013); no emotions (and/or brain regions regarding to emotions) could be activated during expectation of neutral stimuli. In this case, congruent compared to incongruent neutral faces may have no differences in encoding, resulting that the cognitive tasks during expectation do not modulate the congruency effect with respect to face encoding.

While congruent fearful faces are facilitated in terms of face encoding, incongruent faces are related to increased attentional resources. This modulation of emotional congruency on attention could be reflected in the P3. The P3 is thought to represent attentional processes (Hajcak et al., 2006; Olofsson et al., 2008). Many studies have reported that the P3 is more positive in amplitude for incongruent compared with congruent stimuli (e.g., Delplanque et al., 2005; Volpe et al., 2007), indicating that incongruent as compared to congruent stimuli capture more attention (e.g., Schröger et al., 2007; Vachon et al., 2012). Therefore, our data may imply that cognitive tasks alter the difference between incongruent and congruent faces in attention capture.

As several aspects of expectation processing (i.e., probability, violation of expectation, novelty, and cognition) are related to the congruency effect in attention capture (e.g., Polich, 1990; Friedman et al., 2001; Simons et al., 2001; Martens et al., 2006; Steinbeis et al., 2006; Schröger et al., 2007; Vachon et al., 2012), the reason for the modulation of cognitive tasks on the congruency effect may be that cognitive tasks decrease these aspects of expectation processing. Probability estimation of stimulus occurrence is thought to modulate attention, with more attentional resources for stimuli which occur infrequently as compared to frequently (e.g., Polich, 1990; Martens et al., 2006). Therefore, cognitive tasks may prevent probability estimation during the expectation phase and thus, alter the congruency effect to facial expressions. In addition, individuals may prepare for the upcoming facial expression according to the cue during the expectation phase. Violation of expectation is supposed to enhances attention to re-adapt to the stimulus (e.g., Steinbeis et al., 2006; Schröger et al., 2007; Vachon et al., 2012). Therefore, cognitive tasks may reduce the preparation during the expectation phase and thus, decrease the congruency effect.

In addition, our findings showed that cognitive tasks during expectation generally reduced P3 amplitudes to faces. The finding may be in line with previous studies in which cognitive tasks were performed during stimulus presentations (MacNamara et al., 2011, 2012; Van Dillen and Derks, 2012). In these studies, the P3 (in some studies also referred to as LPP) to (emotional) stimuli (e.g., faces and pictures) was found to be smaller under high compared to low cognitive load. However, Erk et al. (2006) did not find an effect of cognitive tasks during expectation on the processing of the upcoming stimuli. The discrepancies between Erk et al. (2006) and our findings may be related to the presentation duration of the stimuli. The duration was long (i.e., 7920 ms) in Erk et al. (2006), but short in ours (800 ms). Previous studies indicate that cognitive tasks during presentations of (emotional) stimuli decrease the processing of the stimuli (Ochsner and Gross, 2005; McRae et al., 2010). However, when cognitive tasks were performed prior to the stimuli, the modulation of cognitive tasks may be reduced. Such modulation may be even smaller when the presentation duration of the stimuli is long, as long duration may allow participants to have enough time to process the stimuli. Therefore, the modulation of cognitive tasks on the upcoming stimuli was not evident in Erk et al.'s (2006) study. In line with our assumption, a study by Iida et al. (2012) found the effect of prior cognitive tasks (though not during expectation) on the stimuli when the stimulus duration was rather short (about 1000 ms).

Consistent with previous studies in which cognitive tasks and expectancy congruency are not included (i.e., Batty and Taylor, 2003; Schupp et al., 2004; Lin et al., 2015), we found that the N170 and the P3 were generally larger for fearful compared to neutral faces. The findings might be also in accordance with MacNamara et al.'s (2012) study in which cognitive tasks were performed during presentations of (fearful and neutral) facial expressions. Fearful compared to neutral faces were found to enhance N170 and P3, regardless of cognitive load. Using a paradigm similar to MacNamara et al.'s (2012) study, however, Van Dillen and Derks (2012) found that while the emotional effect on the P2 (which is thought to have the same brain generators as those of N170; Joyce and Rossion, 2005) was not influenced by cognitive load; this was the case for the P3 effect. The discrepancies among MacNamara et al.'s (2012), Van Dillen and Derks's (2012) and our findings may be related to different facial expressions. Fearful and neutral faces were used in MacNamara et al.'s (2012) and our study, while angry and happy faces were used in Van Dillen and Derks's (2012) study. The categories of facial expressions may be a potential factor which alters the modulation of cognitive tasks (load) on emotional P3 effects.

While the present study found that cognitive tasks during expectation modulated the congruency effects to facial expressions in ERPs, these results were obtained from a cognitive task which was quite demanding. It is unknown whether similar findings will be also observed in a task which is less demanding. Therefore, future studies should be devoted to investigate whether the amount of the task (e.g., load) during expectation modulates the ERP effects of emotional congruency. In addition, studies using S1–S2 paradigms indicate that expectation occurs in two stages of processing: the first stage appears 1.5–2 s after the onset of S1 and is related to the alerting and attention properties of S1, while the second stage appears about 500 ms before the onset of S2 and is associated with the motor response preparation to S2 (e.g., Rohrbaugh et al., 1976; Sakamoto et al., 2009; Lin et al., 2014). The present study suggests that cognitive tasks during the first processing stage of expectation modulates the ERP effects of emotional congruency, while it remains unclear whether such ERP effects could be modulated by cognitive tasks during the second processing stage of expectation. In future studies, we hope to investigate whether cognitive tasks shortly before the occurrence of the face modulates the ERP effects of emotional congruency.

## Conclusion

Our findings revealed that cognitive tasks during expectation modulated the congruency ERP effects to facial expressions. For fearful faces, the congruency effect was modulated by cognitive tasks in N170 amplitude; congruent as compared to incongruent fearful faces evoked larger N170 amplitudes in the non-task condition but such a congruency effect was not evident in the task condition. Regardless of facial expression, cognitive tasks during expectation also altered the congruency effect in P3 amplitude; incongruent as compared to congruent faces elicited larger P3 amplitudes in the non-task condition but such a congruency effect was not shown in the task condition. Taken together, the findings indicate that cognitive tasks during expectation reduce the processing of expectation and subsequently, alter the congruency ERP effects to facial expressions.

## Author contributions

TS and CS were involved in study design, data analysis and manuscript revises. HL was involved in study design, execution, data analysis and manuscript drafting and revises. We have read and approved the manuscript and agree to be accountable for all aspects of the work in ensuring that questions related to the accuracy or integrity of any part of the work are appropriately investigated and resolved.

### Conflict of interest statement

The authors declare that the research was conducted in the absence of any commercial or financial relationships that could be construed as a potential conflict of interest.
